# The challenges and mental health issues of academic trainees

**DOI:** 10.12688/f1000research.21066.1

**Published:** 2020-02-11

**Authors:** Renee Eleftheriades, Clare Fiala, Maria D. Pasic

**Affiliations:** 1 Department of Pathology and Laboratory Medicine, Mount Sinai Hospital, Toronto, ON, Canada; 2 Laboratory Medicine and Pathobiology, St. Joseph’s Health Centre, Toronto, ON, Canada; 3 Department of Laboratory Medicine and Pathobiology, University of Toronto, Toronto, ON, Canada

**Keywords:** academic training, mental health, trainee challenges, anxiety, depression, graduate students, academic jobs, mindfulness

## Abstract

In the last decade, mental health issues have come to the foreground in academia. Literature surrounding student mental health continues to grow as universities try to implement wellness services and study the mental health of their students. Studies vary greatly in terms of measurement tools, timeframe, sample demographics, as well as the chosen threshold of symptom severity for diagnosis. This review attempts to summarize, contextualize and synthesize papers that pertain to the challenges faced by academic trainees at the undergraduate, graduate and post-graduate level.

The evidence for, and against, the common claim of increasing prevalence of mental health issues among students in recent years is discussed. While some studies support this claim, it is difficult to reach a definitive conclusion due to numerous confounding factors such as increased help-seeking behaviour, greater awareness of mental health issues and weak methodology. The prevalence of depression, anxiety, suicidal and self-injurious behaviour, distress and general mental illness diagnoses are discussed. Other issues known to influence mental health, such as sexual assault and bullying, are briefly addressed.

Finally, select studies on a few wellness strategies that may improve mental health of trainees, such as mindfulness, are summarised, along with diverse recommendations for individual students, universities, and academia as a whole.

## Introduction

In this review paper, we discuss the complex issues facing academic trainees and use many abbreviations summarized in Table 1 (references 1– 14). Today, the pressure faced by university students at all levels may be greater than it has ever been in the past 25 years ^15^. Increasing financial stressors since the 2007 recession are affecting undergraduates and graduates alike ^15– 18^. Students are increasingly concerned with getting a high-status job and earning a high income, and are consequently taking their academics more seriously ^15, 19– 21^, resulting in increased feelings of academic pressure as early as high school ^15, 19^. This increased cultural focus on academia is reflected by the fast-paced growth in the number of PhD trainees, which is now outpacing the growth rate of the academic employment market ^22^. As their numbers increase, graduate students face greater competition for a tightening pool of academic jobs, funding resources and mentorship ^16, 23, 24^. These raised stakes underscore the pre-existing stresses and high competition inherent to academic culture ^25^, resulting in intensely stressful conditions ^26^. Thus, it is unsurprising that students have been repeatedly found to have worse overall mental health compared to the general population, characterized by elevated rates of depression, distress and other mental illnesses ^27– 31^.

Several studies have posited that the prevalence and severity of university student psychological issues is on the rise according to counselling centres, self-reported mental health data and symptom severity scales ^3, 32, 33^, though the validity of these findings has been debated ^34, 35^. Still, even though the exact trends in psychopathology are difficult to verify due to confounding factors, mental health is increasingly being recognized as an important issue. The Cooperative Institutional Research Program at Higher Education Research Institute (CIRP) of the University of California at Los Angeles found that, for incoming freshmen, “student self-rating of emotional health was at an all-time low and feeling overwhelmed in high school had been increasing” in 2011 ^15^. In tandem with recent social movements to reduce stigma around mental illness and promote awareness, many universities are now trying to incorporate wellness into the student experience. This review will discuss some of the literature relevant to a variety of student mental health issues and some of the proposed solutions therein. Abbreviations used in this review can be found in Table 1.

**Table 1.** Abbreviations used in this review.

Abbreviation	Description	Reference (if applicable)
AUU	American Association of Universities	
ACHA NCHA	American College Health Association National College Health Assessment	
BDI	Beck Depression Inventory	2
CBT	Cognitive Behavioural Therapy	3
CES-D	Center for Epidemiological Studies Depression	4
CIRP	Cooperative Institutional Research Program	
CORE-OM	Clinical Outcomes in Routine Evaluation-Outcome Measure	5
DASS-42	42-item Depression, Anxiety, and Stress Scale	6
GAD-7-7-item	Generalized Anxiety Disorder scale	7
GAIN-SS	Global Appraisal of Individual Needs-Short Screening	8
GHQ-12	12-item General Health Questionnaire	9
GYA	Global Young Academy	
HERI	Higher Education Research Institute	
K10	10-item Kessler scale	10
MDI	Major Depression Inventory	11
MEES	Mundane extreme environmental stress	12
MMI	Mild-moderate mental illness	
NSSI	Non-Suicidal Self Injury	
OECD	Organisation for Economic Co-operation and Development	
PHQ-9	9-item Patient Health Questionnaire	14
SMI	Severe Mental Illness	
SCOFF	A screening tool for eating disorders	1
RAINN	Rape, Abuse, and Incest National Network	

## Are student mental health issues increasing in prevalence and complexity?

There is growing evidence suggesting that the prevalence and/or severity of university mental health issues has been increasing over the past half-century. Summarized in Table 2, this section will discuss some of this evidence, as well as its criticisms. Firstly, as discussed in the Introduction, the CIRP at the University of California, Los Angeles, asserted that freshman self-reported mental health is at an all-time low for the past 25 years, as a direct result of increasing economic pressures ^15^. The study found that this deterioration in self-reported mental health coincides with an increase in student loan use and a decrease in financial independence due to decreasing job opportunities.

**Table 2.** Summary of the topics covered in this paper. Please refer to Table 1 for abbreviations.

Topic	Description	Assessment scales
Depression	Depressive symptom scales assess feelings of sadness, hopelessness, self-blame, emptiness, disturbances in sleeping and appetite and concentration difficulties.	BDI, PHQ-9, CES-D, MDI, DAS-42, Goldberg
Anxiety	Anxiety symptom scales assess feelings of nervousness, worry, restlessness, dread, etc.	PHQ-9/GAD-7, DAS-42, Goldberg
Distress	Psychological distress is a general measure of negative feelings, stress, mood dysphoria, and psychological strain. Distress is positively correlated to the likelihood of developing a mental illness ^23^.	DAS-42, Trauma Symptom Inventory, GHQ-12
Suicidality	Includes suicidal thoughts, consideration, ideation, planning, or attempts	GAIN-SS, Meehan Inventory

Twenge *et al*. ^33^ performed a cross-temporal meta-analysis of self-reported student mental health between 1938 and 2007. The authors chose to do a cross-temporal analysis to avoid the issue of response bias which results from people with depression not living as long as the average population. They also controlled for each generation’s propensity to lie and corrected their answers on psychopathology scales, adjusting for newer generations being more open to answering the survey honestly. Their assessment of mental health via the Minnesota Multiphasic Personality Inventory found that mental health worsened in each generation, including increased anxiety, depression, and dissatisfaction. These authors attribute this worsening to increased materialism and ambition. They comment that such ambition may be unrealistic, leading to eventual depression, and also that these goals may not be as fulfilling finding “meaning” in life and building interpersonal relationships.

Gallagher, in his National Survey of College Counselling Centers, reports that 94% of American college counselling centre directors agree that there is a continuously increasing number of students with severe psychological problems ^36^. Almost 90% of directors reported an increase in anxiety disorders over the past 5 years, while 58% reported an increase in clinical depression. It is also worth noting that 69% “reported an increase in crises requiring immediate response”, such as a student with a suicidal ideation.

It is entirely possible that this increase in students seeking counselling is due to the reduction of social stigma around mental health. Hunt and Eisenberg ^35^ explore this possibility and find that this increased prevalence in reported problems does coincide with increases in help-seeking behaviour, but that it is possible that mental illness severity is indeed increasing in tandem.

Benton *et al*. ^3^ performed an analysis of the severity and frequency of the problems presented to campus therapists between 1988 and 2001, using the Case Descriptor List, a report filed by the therapist at the end of the client’s case. They found a threefold increase in suicidal tendencies, a twofold increase in depression, and a fourfold increase in issues stemming from sexual assault, concluding that not only the prevalence, but also the severity, of student mental health problems were on the rise. Until 1994, relationship issues were the most common reason for seeking counselling, but thereafter, depression and anxiety took its place. However, they acknowledged that several studies using client self-report data or “objective” measurement scales (i.e. not dependent on the report of the client or psychologist) consistently find no such increase. This raises the possibility that, for unknown reasons, therapists perceive an increase in student issues that is not reflected by self-reports or other attempts based on objective measurements.

In fact, based on this possibility, there is a body of work disputing the claim that student mental health issues are increasing at all. Sharkin disputes the validity of using counselling practitioners’ perceptions as evidence for an increase in mental health issues ^37^. He also offers a critique on the aforementioned Benton study, firstly on the basis of its methods of measuring distress, which he feels are too vague to produce conclusive results, and secondly on its potential sampling bias, since it only examined students already receiving counselling ^34^. However, it is important to note that Benton *et al*. did find a likely-genuine increase in students seeking counselling for career-planning issues. The authors believe this increase to be objectively genuine (rather than an artefact of therapists’ perceptions) because the number of students seeking vocational counselling decreased upon the establishment of a career-development centre at the studied university, but then increased again later ^3^. This increase in career concerns is consistent with that described by the CIRP ^15^, Twenge *et al.* ^33^, and Bishop ^19^.

Costello and colleagues found no difference in depression levels for young persons over a 30-year span in a review of 26 studies, although cohorts were not specific to university students ^38^.

Strepparava and colleagues performed a 5-year study of Italian students receiving counselling, but they did not find an increase in mental health issue severity or prevalence ^39^. However, 5 years is a very short timeframe for assessing overarching trends in mental health issues ^37^.

Cornish *et al.* ^40^ studied university students over a 6–8 year span and found no significant increase in mental health problems, and Berger *et al*. ^41^ and Franke *et al*. ^42^, actually found a decrease in mental health issue severity over time. However, the latter two authors were both studying German students, and attribute the decrease to positive changes in academic conditions that are specific to Germany only (for example, massive reduction in tuition). Furr *et al*. also found that depression rates decreased from 81% to 53% between 1987 and 2001 ^43^. Pledge *et al*. acknowledge a large body of works that have found increases in mental health issues up to the 1980s, but the authors themselves did not find any difference between 1989 and 1995. They hypothesize that mental health issues may have been increasing until the 1980s, but thereafter began to plateau ^44^. Finally, in a systematic review of 24 studies, Ibrahim and colleagues found no trend of increasing prevalence of mental health problems between 1990 and 2010 ^27^.

Thus, the trend in student mental health issue prevalence is a controversial topic. While the above studies may not be reflective of a true increase in mental health issue prevalence among students, they reflect the importance of well-staffed and well-resourced university counselling centres and reveal that mental health and wellness are increasingly becoming important to students and universities.

## Depression

Rates of depression reported among students vary greatly, depending on factors such as symptom scale, demographics and time period, as summarized in Table 3. This section will discuss various reports and contextualize their findings. The American College Health Association National College Health Assessment (ACHA NCHA) Fall 2018 ^45^ reported that 17.3% of respondents (a mixed sample of about three quarters undergraduates and one quarter graduates) had accessed professional services for depression in the past 12 months.

**Table 3.** Representative depression rates among undergraduate and other students. Please refer to Table 1 for abbreviations.

Source	Measurement scale	Demographic	Time range of symptoms	Type and result
ACHA NCHA ^45^	Diagnosis or treatment by professional	Mixed: ~85% undergraduate; 15% graduate sample	Past 12 months	17.3%
Ibrahim *et al.* ^27^	Various, including BDI, CES-D, and PHQ-9 (literature review)	Global, undergraduates	N/A	10-84.5% Weighted mean: 30.6%
Eisenberg *et al.* ^17^	PHQ-9	Mixed: 74% undergraduate; 26% graduate sample	Past 2 weeks	Any, 17.3%; Major, 9%; Mild, 8%
Zivin *et al.* ^48^	PHQ-9	American undergraduates	Past 2 weeks	Any, 13- 15%
Zivin *et al.* ^48^	PHQ-9	American undergraduates	Past 4 weeks	Any, 15.4%
Eisenberg *et al.* ^47^	PHQ-9	Undergraduates and graduates	Past 4 weeks	U, 13.8%; G 11.3%
Farrer *et al.* ^49^	PHQ-9	American undergraduates	Past 2 weeks	Major, 7.9%
Garlow *et al.* ^50^	PHQ-9	Emory University undergraduates	Past 2 weeks	Mild, 29.6%; Moderate, 30.6%; Severe, 6.6%
Gress-Smith *et al.* ^51^	CES-D	American undergraduates	Past week	Any, 33.5%; Moderate- severe, 14.5%; Mild, 19%
Bayram & Bilgel ^52^	DASS-42	Turkish undergraduates	N/A	Moderate, 27.1%
Boujut *et al.* ^20^	Varying (literature review)	French students	Past 12 months	Any, 30%; Major, 6%
Boujut *et al.* ^20^	BDI	French freshmen	Past week	Moderate or severe, 18%

Systematic literature reviews have found massive ranges of depression incidence among students: Ibrahim *et al.* found a global range of 10-84.5% among undergraduates ^27^ and Alfaris *et al.* found 8–70% ^46^ among Saudi Arabian health science students specifically. These ranges include the extremes of the most well-to-do universities in rich regions, to highly competitive conditions in impoverished regions. The weighted mean global rate of depression found by Ibrahim *et al.* was 30.6% ,which is closer to the following studies discussed in this review (though the mean for non-medical students only was slightly higher, 35.6%).

Independent studies surveying student symptoms using the PHQ-9 (Patient Health Questionnaire 9) for depression reported 13.8% ^47^, 17.3% ^17^, 15.4% ^48^, and 7.9% ^49^ among American, and in the last case, Australian, students. A well-known 2007 study on Emory University undergraduate students found the highest depression rates among the PHQ-9 studies included in this paper; they reported that 29.6% of the respondents had mild depression, 30.6% had moderate depression and 6.6% had severe depression ^50^. A study using the CES-D (Centre for Epidemiologic Studies depression) scale found 14.5% for the rate of students with severe depression and an additional 19% with mild depression symptoms, totalling to 33.5% of students having any form of depression ^51^. Using the DASS-42 (Depression, Anxiety and Stress) scale, Bayram and Bilgel found a rate of 27.1% for depression among Turkish university students ^52^. When Goldberg’s Anxiety and Depression scale was used in a Spanish study, a much higher rate was found—55.6% ^53^. Boujut *et al.* report, in a review of French literature, depression rates of about 30%, with 6% of students suffering from a major depressive episode. In their own study on university freshmen, they reported that 18% scored as moderately-to-severely depressed using the BDI (Beck Depression Inventory) ^20^.

Many studies have also examined the prevalence of general feelings of depression or sadness among students, rather than depressive symptoms on a clinical scale. In a study on Australian first-year undergraduates, Hussain *et al.* found that 21.3% reported feeling “often/always unhappy”, and 9% often/always felt they had nothing to look forward to. However, this study also found that only 8% of first-year students had a diagnosis of either anxiety or depression ^54^. It is worth noting that this study was performed in a rural area, so perhaps such settings have lower rates of mental health diagnoses for a number of possible reasons, including lower access to mental health services. Similarly, a UK study reported that 32% of undergraduate students felt “often or always” down/depressed in the past 4 weeks ^55^. An American study found that 53% of students reported feelings of depression since having started college, but “depression” here was not given a standard definition by the authors but rather left up to the interpretation of the individual respondent. It is also worth noting that grades were the most common reason cited by students for their depressive feelings ^43^.

## Anxiety

The ACHA NCHA Fall 2018 survey reports that 22.1% of a mixed student sample (about three quarters undergraduates and one quarter graduates) had been seen by a professional for diagnosed anxiety in the past 12 months ^45^. In total, 62.3% reported having felt “overwhelming anxiety” in the past year, with 29.5% having felt that way in the past 2 weeks alone. The lowest rates of anxiety discussed in this paper were found by Zivin and colleagues, using the PHQ-9 (GAD-7) diagnosis scale: they reported rates of approximately 4% ^56^, 9.8% ^17^ and 5–7% ^48^. Interestingly, an Australian study using GAD-7, the extended and updated version of the PHQ-9, found a significantly higher rate of 17.5%. The authors explain that this rate may be higher than that found by papers such as Zivin *et al*’s because the extended and thorough nature of the GAD-7 makes it a more sensitive tool for detecting anxiety than the PHQ-9. Since this rate is much closer to that of students found to have been professionally diagnosed with anxiety by the AHCA NCHA, it is likely that the GAD-7 can identify students that the PHQ-9 would miss ^49^.

Higher rates were found using other scales: two papers, one using the Goldberg’s Anxiety and Depression scale and one using the DASS-42 scale, both found rates of 47% ^52, 53^. Please see Table 4 for a summary of anxiety rates among students.

**Table 4.** Representative anxiety rates among undergraduate and other students. Please refer to Table 1 for abbreviations.

Source	Measurement scale	Demographic	Time range of symptoms	Type and result
ACHA NCHA ^45^	Diagnosis or treatment by professional	Mixed: U, ^∼^85% undergraduate; 15% graduate	Past 12 months	22.1%
Eisenberg *et al.* ^56^	PHQ-9	Undergraduates and graduates	Past 4 weeks	Generalized anxiety, 4.2%; Panic disorder, 3.8%
Eisenberg *et al.* ^17^	PHQ-9	Mixed: 67% undergraduate; 33% graduate	Past 2 weeks	Any, 9.8%; Panic disorder, 4.1%; Generalized anxiety, 7%; Both, 1.32%
Zivin *et al.* ^48^	PHQ-9	Mixed: 48% undergraduate, 52% graduate sample	Past 4 weeks	Generalized anxiety, 4.75%; Panic disorder, 6.97%
Farrer *et al.* ^49^	GAD-7	Australian undergraduates	Past 2 weeks	Generalized anxiety disorder, 17.5%
Galindo *et al.* ^53^	Goldberg	Spanish undergraduates	Past week	Any anxiety disorder, 47.1%
Bayram & Bilgel ^52^	DASS-42	Turkish undergraduates	Past week	Moderate to severe, 47.1%

## Other mental health concerns

Some literature does not specifically test for depression or anxiety but rather addresses the prevalence of general mental health concerns among subjects, as summarized in Table 5. In a selection of UK student surveys, 10–20% report having a mental health issue ^55, 57– 59^. In a 2010 study of Australian undergraduates, 19.2% qualified as at-risk for a serious mental illness according to the K10 (Kessler) scale, and a further 64.7% show sub syndromic symptoms that classify them as at-risk of a mild/moderate mental illness. In a comparative age-matched sample of the general population, only 3% classified as at-risk for a serious mental illness, supporting the conclusion that students have significantly higher rates of mental distress compared to the general population ^31^.

**Table 5.** Mental health issues and psychological distress rates among undergraduate and general student samples. Please refer to Table 1 for abbreviations.

Source	Measurement scale	Demographic	Time range of symptoms	Type and result
Stallman ^31^	K10 scale	Australian mixed: 74% undergraduate; 26% graduate	Past 28 days	SMI risk, 19.2%; MMI risk, 64.7%; No risk, 16.1%
Zivin *et al.* ^48^	PHQ-9, SCOFF	American mixed	Depression, anxiety or eating disorder: At any given timepoint: >50% At both timepoints: >33%	
Bruffaerts *et al.* ^60^	GAIN-SS	Belgian freshmen	Past 12 months	Any mental health problem, 34.9%
Marcotte & Lévesque ^25^	Varying (literature review)	European students	Varying (literature review)	Distress, 21-31%
Bírá *et al.* ^61^	GHQ-12	Hungarian health students	Past week	Distress, 19%
Bayram & Bilgel ^52^	DASS-42	Turkish undergraduates	Past week	Distress, 27%
Adlaf *et al.* ^29^	GHQ-12	Canadian undergraduates	N/A	Elevated distress, 30%
Marcotte & Lévesque ^25^	Varying (literature review)	European students	N/A	Psychological distress, 21-31%
Bernaras *et al*. ^5^	Various, CORE-OM	Spanish students	N/A	Clinical cut-off for various distress scales: 47.4%; 63.8%
Rosenthal & Wilson ^62^	Psychological Distress Scale	American undergraduates	Past 2 months	Clinically significant level of distress, 9%
Pereira *et al.* ^57^	Diagnosis	UK students	Ever	Mental health issue, 20%
Kerr ^58^	Diagnosis	UK students	Ever	Mental health issue, 10%
Neale *et al.* ^55^	Self-report	UK students	Ever	Mental health issue, 12%

Using the PHQ-9 and SCOFF eating disorder scale, Zivin *et al.* measured student mental health at the beginning and end of a two-year period. They found that, at either given time point, 35% of students had some probable mental health issue (depression, suicidal thoughts, self-harm, anxiety, or an eating disorder) ^48^. A very similar result was found by Bruffaerts *et al.*, who measured freshman mental health with the WMH-ICS (World Mental Health International College Student) survey and found a prevalence rate of 34.9% ^60^.

Many other studies also examine the prevalence of elevated or clinically significant distress among students. A brief review by Marcotte and Lévesque reports a range of 21–31% among European literature ^25^. Two additional studies included in the present review have found rates that are in-line with this range using the GHQ-12 questionnaire: 19% among Hungarian health students ^61^, and 30% among Canadian undergraduates ^29^. Much higher rates were reported by studies using measures other than the GHQ-12, except for one that used the DASS-42 scale and found a rate of 27% ^52^. Bernaras and colleagues reported that 47.4–62.8% of students were above the clinical cut-off in various measures of distress using the CORE-OM (Clinical Outcomes in Routine Evaluation-Outcome Measure) scale ^5^.

Rosenthal and Wilson reported an even higher prevalence of 83% for “moderate-to-severe” distress among undergraduates, using the Dysphoria Domain of the Trauma Symptom Inventory and Psychological Distress Scale, although only 9% of participants were classified as having clinically significant levels ^62^. Finally, Hyun and colleagues reported that 44–45% of graduate students at University of California Berkeley self-reported a significant emotional or stress-related mental health concern ^63, 64^.

## Suicidality and self-Injury

Suicidality can be classified into consideration, ideation, plans and attempt. Table 6 provides an overview of the discussed studies. Some studies that have made an effort to distinguish serious thoughts from ideation include Eisenberg *et al.*’s ^17^ and Garlow *et al.*’s ^50^, which reported 6.3% and about 10% of students had such thoughts within the year the study was performed. Furthermore, Garlow also found that 19.2% of the 10% group were currently suicidal at the time of the study. Overall, 16.5% of the entire student population reported either a suicide attempt or non-suicidal self-injury incident at any point in their lives. They found that 12% of participants had reported a suicidal ideation in the past 12 months ^45^, 4.2% reported ideation currently often or always ^54^, and 8.5% reported it since starting college ^43^.

**Table 6.** Rates of suicidal and self-injurious behaviour among undergraduate and general student samples. Please refer to Table 1 for abbreviations.

Source	Measurement scale	Demographic	Time range of symptoms	Type and result
ACHA NCHA ^45^	N/A	Undergraduates	Past 12 months	Consideration, 12%; attempt, 2%; NSSI, 8.5%
Eisenberg *et al.* ^65^	National Comorbidity Survey Replication	American mixed	Past year	Serious thoughts, 6.3%
Garlow *et al.* ^50^	N/A	Emory University undergraduates	Past year	Serious consideration, ~10%; Attempt or NSSI, 16.5%
Hussain *et al.* ^54^	N/A	Australian freshmen	N/A	Ideation, at least sometimes, 16.6%; Ideation, often/always, 4.2%; NSSI, 3.7%
Furr *et al.* ^43^	N/A	Almost purely undergraduates (95%), American	During university	Ideation/thoughts, 8.5%
Boujut *et al.* ^20^	N/A	French, varied ages	Varying (since starting studies or in past 12 months): Ideation, 9–15% Ever: Attempts, 3–10%	
Boujut *et al.* ^20^	BDI	French freshmen	Past week	Plans, 4.3%

According to the ACHA NCHA, 2% of undergraduates have attempted suicide in the past 12 months ^45^. Boujut *et al.* reported a range of 9–15% for suicidal ideation and 3–10% for suicide attempts among students ^20^. In total, 4.3% of French freshmen reported having suicide plans. Hussain and colleagues further reported that 3.7% of first years surveyed had thoughts of self-harm (often or always), and that the percentage of students who had attempted self-harm at some point in their lives were 17% female and 11% male ^54^. For undergraduates, the rate of self-harm was 8.5% and the rate of suicide attempts was 2% in the past 12 months ^45^. For pure-graduate groups, the non-suicidal self-injury (NSSI) rate was 7.3% in the past 4 weeks ^66^.

## Graduate students and post-doctorate challenges

Graduate students face additional academic pressures, having to juggle a myriad of responsibilities, such as attending courses, managing their projects and writing papers. Many graduate students feel unsure about their future career paths and are afraid that they will not find a permanent position in academia ^67^. Surveys find that graduate students’ greatest concerns are their work-life balance, career security and success, financial security, and general uncertainty about their professional path ^68, 69^.

As mentioned in the introduction, academic positions and funding are becoming increasingly more competitive ^23, 24^. The Organization for Economic Co-operation and Development reported that in the last 20 years, the number of young PhDs has doubled and academic positions did not keep pace with this rapid growth ^22, 70– 73^. A 2014 report by the National Science Foundation found that employment across the science and engineering fields was the lowest in the past 15 years. For fields outside of science and engineering, employment was at the lowest in the past 20 years ^73^. An American study from 2014 estimated that, in engineering, there are only enough academic positions for 12.8% of PhD graduates ^71^. Meanwhile, a *Physics World* article, using data from the Institute of Physics, estimated that just 1.7% of the UK postdocs in physics would go on to an academic position ^74^.

Graduate education is widely seen as a secure path to a high-status job, but in reality about a third of graduates do not even believe that their PhD significantly improves their job prospects ^68^. Many are afraid that they will not be able to find a job at the end of their studies, and this stress has been found to contribute to depressive feelings among graduate students ^75^.

This increasing competition is also affecting grant funding: a report in the magazine *Nature* found that funding has not been increasing in pace with the growing number of PhDs. Major grant providers, such as the NIH and the European Research Council, now award grants to less than 20% of applicants, most of whom will be older scientists with more established careers rather than young investigators.

American funding success rates have more than halved since 1980 ^67^. A graduate student survey found that 50% of North American PhD trainees felt surprised by how difficult it was to secure funding ^67^. The increased scarcity of funding and research resources is likely contributing to trainee mental health issues ^23, 30^.

This high competition is usually explained by academia’s emphasis on overwork. Especially in the sciences, academia has created a culture that venerates the idea of voluntary self-sacrifice for the collective advancement of humanity ^76, 77^. As a result, there is a persistent expectation on graduate students and young researchers in general, to overwork themselves ^24, 30, 78– 82^. Many academics “see suffering as a badge of honour”, as University of Derby Psychotherapist Gareth Hughes explains in an interview with *Nature* ^78^. For many graduate students and other early-career researchers, it is not out of the ordinary to work 60-80 hours a week ^79, 81, 83^, or at least be expected to ^84, 85^. One study on graduate students of economics, natural sciences and engineering found that about 55% work 41-60 hours per week, and approximately another 25% work 61-80 hours per week. According to this study, the majority of graduate students work far past the 9-5 hours expected in most jobs ^86^. As Evans *et al.* put it “work-life balance is hard to attain in a culture where it is frowned upon to leave the laboratory before the sun goes down” ^30^. Meanwhile, many graduate students struggle to make ends meet on their stipends alone, despite their long work hours ^69, 87^. The veneration of suffering in academia makes it difficult for graduate students to recognize their distress as a valid mental health issue rather than just the hardship expected in higher education ^88^.

Consequently, several studies report that graduate students experience elevated stress levels, even compared to undergraduate students. The Gradresources survey found that 37% of graduate students experienced “more stress than they can handle” as a direct result of their studies, with a total of 45% reporting more stress than they can handle in general. A 2015 University of Arizona study found that 73% of graduate students reported greater than average stress, with 23% describing their stress level as “tremendous” ^89^. In one survey of PhD students, 62% reported worrying about their work even when they were not currently working ^86^. The ACHA NCHA found that 61.4% of graduate students report greater than average stress, compared to 57% of undergraduates ^45^.

Studies comparing graduate student mental health to the general population’s found them to be at greater than two-to-six times the risk of depression and anxiety compared to the general population ^23, 30, 86^. Specifically, Evans and colleagues found, using the PHQ-9 and GAD-7 scales, that graduate students experienced depression and anxiety at rates of 41% and 39%, respectively. Comparatively, they reported rates of 6% for the general population in other GAD-7 studies ^30^. Of course, the comparison studies are chosen on the basis of using the same measurement scale as the primary study, even though they may have been performed under different conditions. For example, the Evans study has been criticized for using a German sample as comparison rather than an American survey ^84^. Levecque and colleagues ^23^ performed a thorough analysis of Flemish PhD student mental health as compared to control groups from other highly educated demographics (those in the general population, those who are employed, and those who are higher education students obtaining a degree other than PhD) using the 12-symptom scale General Health Questionnaire (GHQ-12). They found that PhD students were roughly 1.5-4 times at the risk of experiencing each of the 12 symptoms of anxiety/depression, compared to the three other groups. Further, 32% of PhD students reported four or more symptoms, which classifies them as being at serious risk of developing depression and other common mental illnesses.

Barreira *et al.* reported that 18% of graduate students in economics, natural sciences and engineering scored at or above 10 on the PHQ-9 scale for exhibiting symptoms of moderate-to-severe depression and anxiety. This score corresponds to severity to such a degree that the symptoms would likely garner a formal diagnosis from a professional, according to the authors. They compare these rates to those for the age-matched general population, which were found in other studies to be 5.6% for depression and about 3.5% for anxiety ^86^. Thus, graduate students were found to be 3–5 times more likely than the general population to experience moderate-to-severe depressive and anxious symptoms.

A brief review ^90^ of seven studies on graduate student mental health showed a range of 43-44.7% for experiencing emotional/stress-related problems, including symptoms of depression. The one exception to this rule was the Gloria & Steinhardt study ^91^ which found a depression rate of only 29% but that 58% classified as “languishing”. One study featured in the review is a 2014 UC Berkeley study that employed the CES-D scale. It found that 47% of PhD students and 37% of Masters students scored within the clinical depression range ^75^. These are similar to the results found by Evans *et al.*, and not significantly higher than the percent of students at risk of mental illness found by Levecque *et al*. This study also corroborates Levecque *et al.*’s finding that higher education students outside of PhD programs, including Masters students, have slightly lower rates of mental illness relative to PhDs.

Another particularly informative study was conducted by Garcia-Williams, Moffit, and Kaslow. It found that more than 50% of the graduate sample reported feelings of anxiety and 95.4% reported “feeling nervous or worrying a lot” within the past 4 weeks. Also, within the past 4 weeks, 34.4% scored moderate-or-higher levels of depression on the PHQ-9, and 44.4% felt hopeless. Most strikingly, 7.3% reported suicidal ideation, 2.3% reported forming suicide plans, and 1.7% had committed NSSI within the past 4 weeks ^66^. For comparison, the ACHA NCHA found an NSSI rate of 3.2% in the past 12 months among graduate students ^45^. Information from this section is gathered in Table 7.

**Table 7.** Rates of depression, anxiety, distress, suicidality, and self-injurious behaviour among graduate students. Please refer to Table 1 for abbreviations.

Source	Issue	Measurement	Demographic	Timeframe	Prevalence
Grad resources ^69^	Elevated stress	Students were asked to indicate whether they experience “more stress than they can handle”	American	“In life”	45%
ACHA NCHA ^45^	Elevated stress	Students were asked to indicate whether they experienced “greater than average stress” in the past 12 months	American graduate and professional students	Past 12 months	61.4%
Evans *et al.* ^30^	Depression	PHQ-9	American, 91.5%; International, 8.5% PhD students, 90%; Masters students, 10%	Past 2 weeks	41%
Evans *et al.* ^30^	Anxiety	GAD-7	American, 91.5%; International, 8.5%	Past 2 weeks	39%
Levecque *et al*. ^23^	Risk of developing psychiatric issue	GHQ-12	Flemish PhD students	“Recent weeks”	32%
Levecque *et al*. ^23^	Psychological distress	GHQ-12	Flemish PhD students	“Recent weeks”	51%
Barreira *et al.* ^86^	Moderate-to-severe anxiety	GAD-7	American Economics PhD students	Past 2 weeks	17.6%
Barreira *et al.* ^86^	Worrying about work when not working	Survey	American Economics PhD students	Always or most of the time	62%
The Graduate Assembly of UC Berkeley ^75^	Depression	CES-D	UC Berkeley PhD and Masters students	Past week	PhD, 47%; Masters, 37%
Garcia-Williams *et al.* ^66^	Moderate-to-severe depression Hopelessness	PHQ-9 Indicating having felt sometimes, often, or always hopeless	Emory University students	Past 4 weeks	34.4% 44.4%
Garcia-Williams *et al.* ^66^	Anxiety	Indicating sometimes, often, or always having felt “intensely anxious or having panic attacks” Indicating sometimes, often, or always having felt “nervous or worrying a lot”	Emory University students	Past 4 weeks	52% 95.4%
Garcia-Williams *et al.* ^66^	Suicidal ideation Suicide plans	N/A	Emory University students	Past 4 weeks	7.3% 2.3%
Garcia-Williams *et al.* ^66^	NSSI	N/A	Emory University students	Past 4 weeks	1.7%
Garcia-Williams *et al.* ^66^	Symptoms of disordered eating	Survey questions about concern with weight and control over diet	Emory University students	Past 4 weeks	46.4%
ACHA NCHA ^45^	Depression	Diagnosis or treatment by professional Indicating having felt “too depressed to function” Indicating having “experienced depression”	American graduate and professional students	Past 12 months	14.8% 32.9% 28.4%
ACHA NCHA ^45^	Anxiety	Diagnosis or treatment by professional Indicating having felt “overwhelming anxiety”	American graduate and professional students	Past 12 months	19.5% 60.5%
ACHA NCHA ^45^	NSSI	N/A	American graduate and professional students	Past 12 months	8.5%
ACHA NCHA ^45^	Suicide attempt	N/A	American graduate and professional students	Past 12 months	2%
Eisenberg *et al.* ^47^	Depression	PHQ-9	American graduate students	Past 4 weeks	11.3%
Eisenberg *et al.* ^47^	Anxiety	PHQ-9	American graduate students	Past 4 weeks	3.8%
Hyun *et al.* ^63^	Emotional/stress-related problem	5-item survey	American and international students	Past 12 months	43–46%

## Do graduate students struggle more than undergraduate students?

Despite the extra responsibilities and pressures faced by graduate students, there is evidence that their mental health is slightly-to-significantly better than that of undergraduates ^66, 92^. For example, the ACHA NCHA found that graduates had slightly lower rates of diagnosed depression (14.8% vs 17.3% in undergraduates) and anxiety (19.5% vs 22.8% in undergraduates) ^45^. Also, the ACHA NCHA graduate reference group showed lower rates of feelings of depression (32.9% having felt “too depressed to function” in past 12 months, 28.4% “experienced depression” in past 12 months ), anxiety (60.5% having felt “overwhelming anxiety” in past 12 months, 16.6% in past 2 weeks), NSSI (3.3%), suicide attempts (0.3% in past 12 months) compared to those in the undergraduate reference group (42.8% and 32.9% for depression, 63% and 31% for anxiety, 8.5% for NSSI and 2% for suicide attempt, all in respective order) ^45^.

In contrast, the ACHA NCHA did find a slightly higher rate of suicidal ideation (past 12 months) among graduates (12.7% versus 12%), and other studies have also found that graduate students may have higher suicidality than undergraduates ^66^. Meanwhile, Eisenberg and colleagues also found slightly lower rates of mental health issues among graduate students; with a prevalence rate of 11.3%, they were 2.5 percentage points less likely to suffer from depression than undergraduates, and almost 10% less likely to suffer from anxiety (3.8% versus 4.1%) ^56^. In Gallagher and Taylor’s report, 80% of the 125 student suicides reported in 2014 were carried out by undergraduates ^93^. Most conclusively, Wyatt and Oswalt directly compared undergraduate and graduate mental health and found that undergraduates are significantly worse-off in that regard ^92^.

These effects may be a result of the contradicting forces of increased pressure and of selection bias: graduate students face more adult responsibilities and higher academic pressure. However, in order to get into graduate school in the first place, students must be able to meet a high standard of academic functioning. It is likely that the slightly lower rates of mental illness among graduates reported by some studies are due to such selection bias. Students with severe mental health issues are less likely to be able to continue to graduate school.

## Medical and health studies students

It has been suggested that medical students and other health science trainees have particularly high rates of mental distress ^25, 46, 84^. For example, a Swedish study using the MDI (Major Depression Inventory) found that medical students have a depression rate of 12.9% ^76^, more than 1.5x the prevalence they found in the general population (7.8%) ^94^. As a result, there is an abundance of mental health studies focussing on these disciplines specifically. A review of global literature, performed by Puthran *et al*., concluded that 28% of medical students worldwide could be classified as clinically depressed ^95^. Notably, this study did not find a significant difference in depression prevalence between medical and non-medical students. Meanwhile, a review on Saudi Arabian health science student mental health by Alfaris and colleagues found a weighted average of 47%, calculated from a range of results between 8–70% ^46^. However, this result may not be representative of those in North America. For example, the authors explain that the lowest end of the range (8%) was from an American study, whereas the highest end (70%) was from a Pakistani study. Finally, the global review of literature by Ibrahim and colleagues actually found a lower prevalence of depression in medical students: 25.6% (calculated from a range of 10.3–59%), compared with 35.6% for non-medical students (calculated from a range of 10–84.5%) ^27^. The authors note that this is contrary to expectation and suggest the explanation that medical students are perhaps better at recognizing their own mental health issues and reaching out for help. It is also possible that selection bias plays a role in this finding as well.

Turning to American studies, a study of Illinois pharmacy school students found that, on the PHQ-9 scale, 31.9% registered as having mild depression, 16.9% as having moderate depression, 2.4% as having moderate-severe depression, and 1.2% as having severe depression. The study also noted that pharmacy students were twice as likely as the general population to report experiencing a depressive symptom ^79^. Dyrbye *et al.* measured burnout among medical students and found a prevalence of 49.6–52.8% using the Maslach Burnout Inventory ^96, 97^. More strikingly, the prevalence they found for suicidal ideation was 11.2% over the past 12 months, though they calculated that, if all non-respondents were assumed to experience no ideation, the lowest possible prevalence was 5.8%. When respondents were asked instead if they had ever considered suicide at any point in their lives, the prevalence rose to just over a quarter. Wallin and Runeson, in another Swedish study, found even higher rates of suicidal ideation: 33% and 44% for students in their first and final years, respectively ^98^.

Dyrbye and colleagues also compiled evidence for and against the claim that medical students experience elevated distress and rates of depression and anxiety compared to the general population. They conclude that the vast majority of studies with upper-year medical students found that they do indeed experience significantly poorer mental health than the general population ^28^. This is summarized in Table 8.

**Table 8.** Rates of depression, anxiety, distress, suicidality, and self-injurious behaviour among medical and health-studies students. Please refer to Table 1 for abbreviations.

Source	Issue	Measurement	Demographic	Timeframe	Prevalence
Dahlin *et al.* ^94^	Depression	MDI	Karolinska Institute Medical University students	Past 2 weeks	12.9%
Dahlin *et al.* ^94^	Suicidality	Thoughts Thoughts Attempt (Meehan Inventory)	Karolinska Institute Medical University students	Past 12 months Ever Ever	5.4% 28.8% 2.7%
Puthran *et al.* ^95^	Depression	Varying (literature review)	Global	N/A	28%
Alfaris *et al.* ^46^	Depression	BDI	Saudi Arabian	Past week	Weighted mean 47% (range, 8–70%)
Ibrahim *et al.* ^27^	Depression	Varying (literature review)	Global	Varying	Weighted mean, 25.6% (range, 10–84.5%)
Hunt & Gable ^102^	Moderate to severe depression	PHQ-9	Illinois pharmacy students	Past 2 weeks	20.5%
Wallin & Runeson ^98^	Suicidal thoughts	Survey questions	Karolinska Institute Medical University students	Ever	33–44%
Wallin & Runeson ^98^	Anxious symptoms	Survey questions	Karolinska Institute Medical University students	Ongoing	12%
Dyrbye *et al.* ^96^	Suicidality	Consideration Attempt (Meehan Inventory)	American University students	Past 12 months; ever Ever	5.8–11.2%; 25.1% 1.9%

## Who is most at risk?

Out of the studies included in this review, those that measured gender differences almost unanimously found that women are at higher risk for certain mental illnesses, such as major depression and anxiety, distress, self-harm, and/or suicidal ideation. One such study of the different trends between male and female undergraduate mental illness, Conley and colleagues speculate that males are more likely to avoid dealing with their emotions (avoidant coping), whereas females are more likely to try to acknowledge their feelings and try to deal with them. While avoidant coping is generally not considered healthy, it has not been conclusively linked to greater psychopathology in males and it could potentially be a better strategy for dealing with the stresses of university. The study suggests that female students’ tendency to focus on their feelings in an attempt to remedy them may actually be exacerbating their distress ^99^. Interestingly, this finding of increasing distress over time among females, but not males, was also found in a separate study on Canadian medical students. In this study, male students started the semester with elevated distress (one standard deviation above the general population average), whereas females started with average distress which increased to the elevated level (one standard deviation above the general population average) by the end of the semester ^100^.

This higher propensity for distress may be somehow correlated with the finding that females have higher financial strain and insecurity. The Wisconsin-HOPE Lab found that females were 9 percentage points more food-insecure compared to males ^101^. Stallman’s study found that females were slightly more likely to report “frequent financial stress” and “constant financial stress”, and less likely to report “no financial stress” as compared to males ^31^. However, the author does not comment upon whether these differences are statistically significant. More conclusively, a UK study by the National Union of Students reported that females are 1.5 times more likely to worry frequently about finances, compared to males ^103^.

Additionally, the Global Young Academy found that women had a slightly more pessimistic view of their career prospects, giving lower estimates of their chances to become a professor or land an academic or teaching position. Overall, women gave their career prospects an average rating of 0.1 lower than males, on a scale of 1-5. Also, women were more likely to report lack of support from superiors (50.6% versus 41.2%) and job rationalization (21.3% versus 13.1%), as well as gender discrimination (17.1% versus 4.6%), as obstacles to their career ^104^. However, the authors warn that an existing sampling bias due to overrepresentation of women in the survey was not corrected, so these values should not be viewed as conclusive.

While male students have lower psychological distress than female students, they comprise a much larger portion of student suicides ^35^. For example, in his 2014 survey of university counselling centres across America, Gallagher reported that 70% of that year’s student suicide victims were male ^105^. The UK Office for National Statistics reported that male higher-education students are almost three times as likely to commit suicide as females ^106^. This is consistent with the general population: while females are more likely to have suicidal ideation and/or attempt suicide, males are much more likely to actually end their lives ^107^. This could in part be due to differences in chosen method: males are twice as likely to use a firearm, whereas females are three times as likely to attempt to poison themselves ^107^. Also, Bernaras and colleagues found that men were at a higher risk of doing “actual harm” to either themselves or others ^5^.

Besides sex differences in the preferred method of suicide, another reason for the higher suicide rate among males is that they are less likely to access mental health services or partake in wellness practices, perceiving a higher stigma around discussing personal problems and seeking professional help ^27, 108^. Gallagher’s 2010 National Survey of University Counselling Centres found that just 35% of student-counselling clients are male ^105^. Bernaras and colleagues even cited this discomfort with disclosing personal issues as an explanation for the gender differences many studies have found in rates of psychological distress and related symptoms. Perhaps this gap is due in part to the fact that women are more likely to openly and honestly report their mental health issues and symptoms on surveys ^5^. Also, Conley and colleagues reported that male students typically have less social support than females ^99^, which may be another factor that discourages them from reaching out to seek help. Finally, studies on undergraduates report that females are significantly more prone to eating disorders ^54, 99^, whereas males are more prone to binge drinking ^54, 109^.

Studies including a racial component also found that racial minority students are at higher risk of distress and other mental health issues. Smith *et al.* found that minority students exhibited worse mental health than white students, even at universities that had a high concentration of students from ethnic minorities ^109^. Eisenberg and colleagues found that white students were less likely to screen positive for major depression on the PHQ-9, compared to any other race, with students identifying as “other race” having the highest likelihood ^17, 47^. They also found that students identifying as multi-racial were more likely to experience major depression, suicidal ideation, and NSSI ^17^. This evidence points to the possibility that students who do not easily fall into a single socially defined racial category experience additional stress. Richardson *et al.* corroborated claims that minority students experience poorer mental health, but interestingly, they found white students to be at higher risk of alcohol dependence than black and Asian students ^110^. Notably, Dyrbye *et al.*’s review of North American medical students revealed no link between ethnicity and mental health ^28^. It is possible that medical students have a higher socioeconomic status overall and therefore medical students from an ethnic minority do not experience more economic challenges than white medical students. Students of colour, especially black students, are less likely to seek mental health services compared to white students ^62, 111– 113^. Moreover, minority students may experience additional race-related stress, in the form of MEES (mundane extreme environmental stress, also called “microaggressions”), isolation, and stereotype threat ^12, 111^.

According to the HOPE Lab, minority students were at a higher risk of food and housing insecurity compared to white students, with the exception of Asian students, who were at the lowest risk out of all the racial categories ^101^. Native American students were at the highest risk for food insecurity (55%), housing insecurity (69%), and homelessness (19%), followed by black students (54%, 55%, and 13%, respectively), and “mixed/other” students (50%, 52%, and 17%). Hispanic students were at a slightly lower risk (47%, 51%, and 10%), followed by Middle Eastern/Arab students (43%, 49%, and 12%). White students had the second-lowest risks (37%, 42%, and 11%), followed by Asian students, who had the lowest risks across the board (36%, 37%, and 7%). Native American and “mixed/other” students experienced the highest prevalence of homelessness ^101^. The elevated risk of homelessness for “mixed/other” students may correlate with their higher risk of psychological distress. In the same vein, the CIRP reports that in America, students at historically black colleges and universities report higher levels of financial insecurity, with one in five students unsure of whether they had the financial means to complete their degree ^15^.

It is likely that the link between ethnicity and socioeconomic status plays some role in the increased prevalence of mental health issues among minority students. Socioeconomic status has been linked to worse mental health ^35, 114, 115^, reduced access to mental health services, and reduced wellness behaviour ^35, 108^. Eisenberg and colleagues found that even growing up in a lower- socioeconomic-status household predicted worse mental health in students ^47^. In fact, a NUS Insight survey reports that 36% of students report that they worry about finances so much that it affects their mental health ^103^. A British study found that self-reported financial stress and debt were strongly correlated to worse overall mental health, including depression, anxiety, stress and alcohol abuse. Interestingly, though, this study did not find that family affluence could predict mental health issues, in contrast to the findings by Eisenberg *et al.* ^110^. Cuéllar and Roberts, therefore, argue that studies exploring the link between racial identity and mental health should also control for the mediating effect of socioeconomic status. In their analysis of survey responses gathered from Latino students, they found that socioeconomic status plays a much more significant role in poor mental health compared to ethnic identity ^114^.

Interestingly, in a study of 1,773 undergraduates across four socioeconomic status groups, Rosenthal and Wilson found no disparities in mental health service utilization between sexes, socioeconomic status groups, or ethnicities ^62^. The authors note that this is contradictory to the prevailing assumptions about service use disparity, but they stress that there are no identifiable errors or statistical anomalies that could have erroneously led to these results.

As may be expected, LGBTQ+ students face additional challenges, unique to their identity. There is evidence that homosexual students experience poorer mental health and higher suicidality compared to heterosexual students ^111, 116^ with bisexual students especially at risk. Kerr, Santurri & Peters found female bisexual students to be 3.1 times as likely to report a depression diagnosis, twice as likely to report anxious and depressive symptoms, and almost five times as likely to report suicidality and self-harm, compared to heterosexual female students. Lesbian students were twice as likely to report a depression diagnosis, five times as likely to report self-harm, and about four times more likely to report suicidality, compared to heterosexual female students. Bisexual women were overall significantly more likely to report depressive and anxious symptoms and suicidality compared to lesbian and heterosexual women ^116^. Meanwhile, Liu and colleagues reported that bisexual students were 1.5 times more likely to report a mental health diagnosis or suicidality than homosexual students, and 3.9 times more likely compared to heterosexual students. Furthermore, they report that over half had experienced suicidal ideation and a quarter had made attempts on their lives ^111^. Eisenberg and colleagues also found that bisexual students were at particular risk of experiencing depression ^47^.

As before, this comes with a significantly increased risk of housing and food insecurity: homosexual students had greater risk than heterosexual students, and bisexual students had the highest risk of all. Bisexual students were about 10–15 percentage points more likely to report food and housing insecurity and homelessness compared to heterosexual students, and 5–7 percentage points more likely than homosexual students ^101^.

Not just students from sexual-orientation minority experience additional distress and needs insecurity, but also students from a gender-identity minority as well. A study of transgender and non- binary students found that 85% reported a mental health issue, with 25% explicitly relating to the challenges of being a gender-identity minority to their poor mental health. Many trans/non-binary students are reluctant to access mental health services due to the risk of landing a transphobic or ignorant counsellor. Slightly more than 25% of respondents described their previous counselling experiences as ambivalent or non-affirming towards trans/non-binary issues ^117^. The obstacles facing gender minorities who need to access mental health services are particularly concerning in light of the fact that they are at much higher risk for mental illness ^30, 111, 117^. Evans and colleagues found that transgender students were 12-21 percentage points more likely to report anxiety compared to cisgender males and females respectively, and 16-22 points more likely to report depression ^30^. Liu and colleagues reported that transgender students were 1.9-2.4 times as likely to report mental health issues and suicidality compared to cisgender students. They have a much higher rate of distress compared even to bisexual students, with two-thirds experiencing suicidal ideation and one-third having made an attempt ^111^. It is additionally concerning that some studies have reported an increase in the prevalence of transphobic bullying at universities ^118^.

A report on LGBTQ+ scientists in the physical sciences may be indicative of some of the factors at play in LGBTQ+ students’ mental health challenges: 18% of LGBTQ+ scientists, and in particular, 32% of non-binary scientists, reported being the target of discriminatory behaviour at their workplace ^119^.

## What stops students from getting help?

While great progress has recently been made in dismantling the stigma around mental health issues, stigma is likely still a primary obstacle standing between many students and the help they need ^119^. Hussain *et al.* interviewed some of the first-year subjects in their study and concluded that stigma is still a concern among students who are considering seeking help. Students mentioned feeling that seeking help might be embarrassing, as they would appear unable to handle academic and social stresses. The authors further suggest that students in rural/small-town settings may feel that there is a higher social risk to seeking help, due to the reduced privacy/anonymity of such settings ^54^.

Similarly, Eisenberg, Golberstein, and Gollust investigated the most common reasons that students who need help do not seek it. In total, 20% cited worrying about what others will think of them if they were in mental health treatment, 10% feared that it would somehow go onto their academic record and 9% feared that their parents would find out. Besides stigma, 32% reported simply lacking the time to seek help, and 8% lacked the finances. A total of 32% thought they would just get better by themselves over time, which may indicate a need for better awareness about the seriousness of mental health issues. It is also noteworthy that 51% said they felt that stress was just a regular part of school, though only 45% indicated that they felt they had no need for mental healthcare. A possible interpretation is that the 51% recognized they had more stress than they could handle, but thought it would be inappropriate to seek help for distressed caused by “normal/mundane” problems such as school ^113^.

Interestingly, Eisenberg and colleagues also found that students’ own internalized stigma of mental illness is a bigger obstacle to seeking help than their perception of public stigma. In comparing students’ own internalized stigma with their perception of public stigma, Eisenberg found large disparities, pointing to the possibilities that students either have an inflated sense of public stigma or are under-reporting their own internalized stigma ^120^. The review by Storrie, Ahern, and Tuckett compiles evidence that the vast majority of students with emotional problems and/or severe distress are not receiving treatment and attributes stigma as a key reason ^121^.

In Gallagher’s report, only 13% of people who had completed suicide had received counselling ^105^, though this may be a case of selection bias: students in treatment would be less likely to resort to suicide. In another study on the persistence of student mental health problems, less than half of the students who had a persistent mental health problem over the two-year span of the study received help within that duration ^48^. In Barreira *et al*.’s study of PhD students, only 27% of students with depression and 21% of students with anxiety were in treatment. Although this could simply reflect oversensitivity of the survey’s screening instruments, only 27% of students who self-reported suicidal thoughts were in treatment, which indicates that a majority of students with mood disorders are not receiving treatment ^86^. Similar numbers were reported by Garlow *et al.,* who found that 84% of students with suicidal ideation and 85% of students who screened as moderately-severely-to-severely depressed on the PHQ-9 were also not receiving treatment ^50^. According to Hunt and Eisenberg ^35^, similar rates of treatment among mentally ill students (less than 25%) were also reported by Blanco *et al*. ^122^, though they also noted that this low rate of treatment is not significantly different from the rate among non-students. There is evidence suggesting that men experience greater stigma around mental illness and are therefore particularly unlikely to seek help ^123^. This also seems to be case for Asian and Latino students ^120^.

## Factors that could contribute to mental health concerns

### Sexual assault

Sexual victimisation has been positively linked to suicidal ideation, depressive feelings, psychological distress, and risk-taking behaviour in university student samples of men and women ^124, 125^. According to RAINN, American university students are in the age range with the highest risk for sexual assault, although female students are at a lower risk than female non-students ^126, 127^.

Estimates of university sexual assault frequency vary greatly. For university students of all genders and degree levels, RAINN cites a sexual assault frequency of 11.2% ^126, 127^.

For female students, Stepakoff’s review found between 13-27% were survivors of sexual assault. Outlier studies estimate rates greater than 50% ^124^. American Association of Universities (AAU) statistics cited by RAINN are firmly within this range, reporting a sexual assault frequency of 23.1% among female undergraduates. The reported frequency for female graduate/professional students is much lower, at 8.8% ^128^. Furthermore, they report that only 1 in 5 female student victims report to law enforcement ^114^, and only 1 in 6 receive assistance from victim services ^127, 128^.

Conversely, male students are much more likely to be sexually assaulted than their non-student counterparts. The AAU’s estimate is 5.4% of undergraduates and 2.2% for graduate/professional students have been sexually assaulted ^127, 128^. Turchik reports a range of 18.5%–31% for rates of unwanted sexual contact in the past academic year ^125^. Furthermore, Turchik reports that the range becomes 34–58% when male students are asked to report incidences since age 16 ^125^. Men are less likely to disclose and seek help for their unwanted sexual experience, due in large part to myths about male rape ^128^.

Thus, it is important to educate male students in particular about their right to report, and to dispel stigma around being a male sexual assault victim. Finally, gender minorities are at an elevated risk of sexual assault and rape, especially gender-queer/gender non-conforming/trans-identifying males ^127, 128^.

A survey of graduate students across the fields of economics, engineering, and natural sciences found that 16% of graduate students have experienced sexual assault at some point during their PhD studies. Specifically, 21% of female students and 13% of male students reported such an experience. Overall, 62.5% of instances were perpetrated by peers whereas 19% were perpetrated by a professor ^86^.

### Bullying

It is well-known that bullying negatively impacts mental health ^118^. Bullying in academia can happen by peers or by authorities (supervisors, professors, mentors, etc.). A common form of supervisor-student bullying is academic exploitation, in which a supervisor takes credit for a student’s work ^129^. In other cases, supervisor-student bullying can include making inappropriate, discriminatory, or derogatory comments ^130^. In all of these cases, students can often feel as if they have no means of recourse, because they fear that their concerns will not be taken seriously by higher-ups, or that antagonizing their supervisor will impact their academic/professional careers ^129– 131^. Thus, it is difficult to comment on the frequency of these issues. The hierarchical nature of higher education makes it difficult for students to report their experiences, either because they are afraid of the consequences, or because they believe their experience is normal ^130– 132^.

Yamada *et al.* investigated bullying in a study of Canadian graduate students ^133^. They reported that overall, 21% of students reported experiencing bullying by their supervisor, though very low percentages of students reported that the bullying was frequent. They found that the most frequent bullying types were “destabilization” (changing goals/responsibilities without informing the student, persistently undermining or demoralizing the student, etc.), which was experienced in some form by 40% of respondents, and “isolation” (ignoring the student, neglecting to give them important information, restricting their non-academic activities), which was experienced in some form by 37% of respondents, and finally, “overwork” (unreasonable pressure or deadlines), which was experienced by 32.9% of respondents. The four most common sub-behaviours were “undue pressure to produce work” (31.7%), “shifting goal posts without telling you” (28.8%), “adding or removal of areas responsibility without consultation” (24.5%), and “threat to personal standing” (26.6%). In terms of authorship issues, 41.3% reported that a supervisor or other faculty member received honorary authorship on their work ^133^.

Finally, in Frank *et al*.’s study of 16 medical schools, up to 63% reported having experienced belittlement by a professor and 71% experienced belittlement by a resident. The rates of students reporting harassment by these groups were 21% and 27% ^134^. Issues between graduate students and supervisors are important to document, as Evans *et al.* found that a student’s relationship with their supervisor has significant impact on their mental health ^30^.

A well-studied form of bullying at the undergraduate level is cyberbullying. Kokkinos *et al*. mention that 1/3 of students reported involvement in bullying through online methods, such as over social media. Their literature analysis reported a rate of 9% to over 50% for reports of student victimization ^135^. Myers and Cowie report that students in fraternities/sororities, women, and LGBTQ+ students are particularly vulnerable ^118^.

The recently identified practice of “mobbing” is another type of bullying that academic culture may particularly lend itself to. Mobbing occurs when a team of colleagues collude to exclude, isolate, and demean a specific target. According to Seguin, universities are particularly unable to deal with this subtle form of bullying because they presume to be objective institutions that are “above” social issues such as bullying. In reality, their anti-harassment resources are unequipped to handle such situations. Issues often get passed off euphemistically as “personality clashes” and victims are left with no real recourse ^136^.

### Financial strain and parenting

The Wisconsin-HOPE Lab found striking amounts of needs-insecurity among American undergraduate students across 66 institutions. The authors report that 36% of students reported experiencing food and housing insecurity in the past month, and 9% were homeless in the past year. Needs insecurity was highest among community college students and marginalized groups ^101^. The higher needs insecurity among marginalized groups is discussed in more detail in the “Who is most at risk?” section of this review.

Perhaps unsurprisingly, needs insecurity was also higher among student-parents. In four-year programs, they were 9 percentage points more likely to be food-insecure and 6 percentage points more likely to be housing-insecure. In two-year programs, these differences were even more exaggerated ^101^. In their studies of medical students, Dyrbye and colleagues found that student-parents are more likely to drop out ^97^ and more likely to screen for depression on the CES-D scale, though debate exists as to whether this elevated depression risk applies to fathers and mothers, or mothers only ^28^.

In the essay “Student-parents and higher education: a cross-national comparison”, Brooks explores the unique challenges of being a student-parent and recommends cultural attitude shifts and institutional supports that are necessary to properly accommodate them. The author cites “the temporal demands of being both a student and a parent of a young child; the paucity of on-site childcare facilities; restrictive ‘no child on campus’ policies; late availability of educational timetables; inconvenient timing of lectures and acute financial pressures” as a few of these challenges, as well as the conflict between their identities and expectations as students versus parents ^137^.

In a *Nature* article, Powell interviewed an American mother who describes the challenges of being a PhD student and early-career scientist in a country with no formalized paid parental leave. This article describes a similar lack of on-campus childcare services and provisions, such as private lactation rooms. It also highlights restrictive conference policies that do not allow speakers or attendees to bring their infants with them ^138^. Ultimately, both pieces point to the problematic expectation that academics must forgo family for career success, leaving student-and-scientist-parents with little support. According to Powell, childbirth is likely to coincide with the most crucial stage of a female early scientist’s career, forcing her to make difficult choices ^138^. The GYA corroborates that scientist-mothers in particular point to work-life balance as an obstacle in their careers, significantly more than childless scientists or fathers ^104^. Their recommendations are covered further in Table 9.

**Table 9.** A summary of institutional and cultural changes recommended by the authors featured in this review.

Source	Issue	Recommendation
Garlow *et al.* ^50^	Student suicidality	Outlines a method used at Emory University: An annual anonymous mental-health- screening survey is sent to students over the internet; students with concerning scores are urged to come for in-person help
Evans *et al.* ^30^	Mental health issues among graduate students	Establishing career development programs that can encourage mental wellness while addressing common career-based concerns among graduate students Educating staff about mental health issues via a “train-the-trainers” method of relaying information among the staff network Encouraging staff to endorse self-care and mindfulness as key to efficient work
Woolston ^139^	High stress levels experienced by graduate students	Establishing/joining a student-run or young- scientist-run community for advice and support, such as the Cheeky Scientist Association Seeking therapy Getting involved with student association events, such as professional- development or leisure activities Encouraging graduate students to be firm and confident in their work-life boundaries Reaching out to other young scientists to show positivity and support Encouraging out-of-lab public engagement activities Broadening the model of acceptable research beyond just outcomes, so that it also includes new research methods Broadening the model of acceptable career paths beyond just tenure-track
Powell ^82^	Lack of funding for young scientists due to competition by older, more experienced scientists	Following the approach of funders such as The European Research Council and NIGMS, which allocates a certain amount of grant money for early-career researches only
Woolston ^81^	Overwork and burnout among graduate students and young scientists	Shifting the focus from strictly the number of hours worked to the amount of worked produced; encouraging students to be efficient and make the best of their time rather than working longer and longer hours Supervisors should be lenient about work hours and allow time for “life”
Powell ^140^	Burnout and overwork among students	Educating students to recognise the signs of burnout in themselves and others Again, focusing on efficiency over hours worked Recognising the importance of taking breaks, which should be short and frequent rather than long and occasional. Breaks should be entirely removed from the setting or topic of work. One interviewee suggests the “Pomodoro method”: taking a 3–5-minute break every 25 minutes of work, and occasionally taking an extended 15–30-minutes break Recognising that happiness is key for good productivity, and treating good spirits as an important work strategy rather than merely an emotional consolation Students would benefit from identifying their most productive time of day and planning their biggest tasks accordingly Using small, easy tasks to break up long, difficult tasks; small tasks can include out-of- lab responsibilities such as household chores Supervisors should be understanding about self- care and student needs
Woolston ^141^	Low self-esteem and isolation among students, with a particular focus on STEM and graduate student issues	Teaching students to recognise and fight Impostor Syndrome feelings; encouraging them to celebrate their accomplishments rather than trivialising them Normalising rejection; understanding that is a healthy part of any academic career Reaching out to other students who are struggling For gender/race minorities, joining an online community of researches in your field who share aspects of your identity Government-instituted community programs for collaboration between young scientists, such as the Participatory Science Platform in New Zealand
Wong ^83^	Students feel alone and/or unsure	Restructuring academia to emphasise and reward faculty mentorship of students
Storrie *et al.* ^121^	Accessibility and efficacy of university counselling services	Better communication between academics, university counselling, and community counselling to create a more organised student- support network Reducing stigma, emphasising confidentiality of mental health services, and raising awareness of available help
Marcotte and Levesque ^25^	Distress and low self-esteem among younger undergraduates	This study explores the relationship between distress and sense of identity, and suggests that this relationship is taken into account by counsellors; it could also possibly be used in mental health screening Helping students develop a solid sense of identity, which the authors show correlates with better mental health Setting up a program in which older students could mentor first-year students to help navigate and normalise identity issues Implementing programs that can contribute towards goal setting and value-formation
Parkman ^142^	Imposter phenomenon feelings and low self-esteem among students at the undergraduate and graduate levels	Recognising the effect of imposter phenomenon on mental health and working to address it in counselling, workshops, and orientations Teaching students how to combat perfectionism and feelings of failure by focusing on goal- setting and positive aspects of their identity
Powell ^143^		Academia has an individualistic “winner takes all” culture, in which one person typically receives all the glory for a scientific breakthrough, or all the blame for a failure. This article highlights the need to shift academia’s perspective from individualistic to collective, one in which academics help each other and work together This could turn academia’s stressful hypercompetitive environment into an open and comfortable one By extension, dismantling the elitism that allows/encourages higher-status academics to be dismissive or rude to younger academics; as ecologist Emily Bernhardt explains it in the article, “There’s this idea that is’ OK to be an awful person as long as you are brilliant”
Goldrick-Rab *et al.* ^101^	Student need- insecurity	Students can get involved in student-run organisations that fundraise to combat need- insecurity and raise awareness Universities should develop aid programs that are private and accessible, and ensure that students know how to use them; or they should consider partnering with existing aid charities
Goldberg *et al.* ^117^	Trans and non-binary students’ reluctance to use mental health services due to prejudiced or ignorant practitioners	Educating counsellors to recognise, but not overemphasise, trans/non-binary issues in the context of counselling Dispelling the narrow conceptualisation of transness among counsellors (i.e. the idea that a patient must meet certain conditions to prove that they are “trans enough”)
Barnett *et al.* ^12^	Under-utilisation of mental health services by black students; unaddressed racial stress experienced by black students	Counselling should incorporate behavior-based theories and strategies that can help black students deal with the duality of the expectations and identities they take on as Black students in a Eurocentric education system The authors also recommend cooking classes that teach students how to prepare healthy foods on a budget, and transition classes to help deal with the change from high school to university

## Wellness

Wellness is a broad concept that endorses a holistic view of health, wherein physical mental, and emotional well-being are equally important. It embraces self-care, stress-relief and management, healthy emotional coping, mindfulness, and reaching out for help when necessary. As such, it emphatically promotes mental-health awareness and stigma reduction, and, more broadly, cultivation of good mental/emotional framework for coping with stress and grievances in a healthy manner ^144^. A key tenet of wellness is the value of prevention over treatment ^145^. Prevention is achieved by prioritizing an individual’s well-being, being aware of one’s health needs and adapting one’s lifestyle accordingly.

Focussing on staying healthy and being aware of signs of illness, whether physical or mental, is a more productive approach to health as opposed to waiting until after one is already sick. Physical wellness include exercising and eating well, emotional wellness can include building a social support group and implementing healthy coping strategies, and mental wellness can include mindfulness activities and counselling, if necessary. Wellness may have been considered unimportant, or even a distraction from work, in the past, but cultural attitudes are shifting, as evidence amasses showing that employee happiness is key to maximizing productivity ^146, 147^.

Several studies have assessed the effectiveness of wellness practices such as meditation, mindfulness, counselling and mindful physical exercise. Conley, Durlak, and Dickson performed an analysis of 83 studies to assess the effectiveness of mental health promotion and prevention programs in higher education. Their review confirms the effectiveness of wellness programs in alleviating depressive and anxious symptoms and highlights cognitive behavioural therapy and mindfulness as the two most effective wellness strategies. The study further concluded that supervised, skill-oriented, class-format wellness programs are the most effective ^148^. In their own experiment with a wellness seminar for first- year students, Conley and colleagues once again found that attendance and engagement with the wellness program are key to seeing improvements. Participants who regularly attended wellness seminars and practiced the skills they were taught showed better psychosocial and academic adjustment and better stress management ^149^.

In another such review, Fernandez and colleagues examined the effectiveness of university mental health promotion and intervention programs, as well as the qualitative validity of the studies being analysed. The topic of the studies analysed varied greatly, so it is difficult to generalize their results here. However, their assessment of the program efficacy, combined with validity considerations, had an overall mixed tone. For example, a 1984 University of Illinois policy mandating students with suicidal ideation to receive four therapy sessions appeared to reduce suicides by 72%, but oddly, graduate/professional student suicides rose by 94.6% following this policy ^150^. A Chinese study on the impacts of a “youth development” wellness course reported that it was effective in increasing positive and hopeful feelings, but Fernandez and colleagues deemed the study to be of questionable credibility due to its lack of detail ^151^. A third study found that subjecting medical students to a stress-management lecture reduced their stress by 46.7%, but again, it was deemed of low-credibility due to its lack of control group. Also, many other studies included did not find that such programs had any significant positive impacts. One repeated finding among studies in the review that did seem conclusive, however, was the impact of grading systems on student health: grading systems with fewer increments seemed to reduce stress, depression, and anxiety ^151^.

Universities are trying a variety of approaches to improve student mental health. For example, a few studies have explored the possibility of using tai-chi to foster wellness among students. One such study found no significant effects on mental health ^152^, but another study found that tai chi chuan significantly decreased anxiety and improved sleep quality among young adults ^153^. In fact, a systematic review of the impact of tai chi on higher-education student mental health concluded that it is an effective method of reducing anxious and depressive symptoms among students ^154^. As mentioned earlier, mindfulness has been a hot topic in student wellness discussions. Vidic & Cherup reported that mindfulness relaxation was so effective among a sample of college students, that the test group went from higher stress than the control group to lower stress ^155^.

In a review of empirical mindfulness studies, Keng, Smoski, & Robins analyse a growing body of credible evidence that general mindfulness practices have a positive impact of mental health ^156^.

However, the two university-sample studies in the review have conflicting findings: *Bluth et al.* found that mindfulness can uplift bad mood in students ^157^, whereas Kuehner, Huffziger & Liebsch did not find any significant impact ^158, 159^. The authors explain that this may be due to differences in sample or in methodology ^156^. Cook recommends the usage of cognitive behavioural therapy and wellness courses to alleviate student mental distress, based on evidence supporting their effectiveness ^119^. Cook references Brown & Schiraldi, who found cognitive behavioural therapy to be significantly more effective in stress-reduction than a conventional stress-management course among students. Similarly, Kitzrow compiles evidence on the positive impact of counselling on both student mental health and academic performance ^32^.

Combining the wellness principles of mindfulness and exercise, Caldwell *et al*. studied the benefits of mindfulness established through movement-based courses for university students. They reported that that such courses were successful in reducing negative arousal and the prevalence of insomnia, but mood changes were only statistically significant through the mediating factor of mindfulness: students who showed an increase in mindfulness (via the Five Facet Mindfulness Questionnaire) also showed an increase in positive feelings, relaxation, self-regulatory efficacy, and sleep quality, and a decrease in perceived stress ^154^. Finally, this paper also includes recommendations from papers and articles on policy and attitude changes that can hopefully make academia a more welcoming and less stressful environment. For convenience, these are presented in Table 9.

## Conclusions

As shown in this review, there is a plethora of studies covering various aspects of student mental health, using diverse measurement tools and consequently, finding a very broad range of prevalence. Even among two studies using the same measurement tool, the reported prevalence of mental illness may differ significantly, depending on what level of symptom severity the authors choose as the cut-off.

Continuing to refine and standardize measurement tools seems necessary. Given the numerous strains and concerns specific to students and trainees in academia, it would seem appropriate that studies should view student wellness within the context of these challenges and continue assessing student mental health in comparison to that of the general population. Such comparisons illustrate whether student-specific challenges contribute to poor mental health, and therefore direct the approach of student wellness initiatives. For example, student wellness initiatives can guide students to deal with student-specific challenges or recommend changes to mitigate these challenges at the institutional level, rather than simply directing students to mental health counselling. Of course, as indicated by even the lower end of the prevalence ranges in this review, ensuring that university mental health counselling is well-staffed, well-resourced and well-educated is paramount to student well-being. This review merely recommends that student mental health be viewed within the context of student-specific challenges and that institutions and institutionalized counselling focus on helping students to deal with these issues.

## Data availability

No data is associated with this article.
