# Supplementary material for: The challenges and mental health issues of academic trainees
Source: F1000Res. 2020 Feb 11;9:104. [Version 1] doi: 10.12688/f1000research.21066.1 (PMC7199285; doi:10.12688/f1000research.21066.1)
Supplement: Peer review history

# Peer review: Reviewer response for version 1

**DOI:** 10.5256/f1000research.23184.r62801

**Published:** 2020-05-04

**Authors:** Leland Ackerson

**Affiliations:** 1 Department of Public Health, University of Massachusetts at Lowell, Lowell, MA, USA

The Introduction of the article points to the 2007 recession as a key event regarding the financial concerns of today’s students. I believe that updating this text to incorporate the Covid-19 related economic conditions will allow this article to remain relevant for a much longer time.

 Some of the cited papers actually diagnosed patients with mental illness (such as depression or anxiety). Others were epidemiologic studies which relied on psychometric tools that provided evidence of symptomology but did not actually diagnose patients. It may be helpful to make this distinction in the current review when relevant. For example, the Garlow *et al.* paper (reference #50) repeatedly refers to “depressive symptoms” while the relevant section of the current review (end of page 5 and beginning of page 6) refers to mild, moderate, and severe depression—not symptomology. I would encourage the authors to use language consistent with the referenced papers.

 Both Liu *et al.* (reference #111) and Kerr *et al.* (reference #116) described sexual minorities as gay/lesbian. Yet on page 14 the authors used the term “homosexual” in the phrase “There is evidence that homosexual students experience poorer mental health and higher suicidality compared to heterosexual students ^111,116^.” This term is outdated and should be replaced in that paragraph and the following paragraph.

Is the review written in accessible language?

Yes

Are all factual statements correct and adequately supported by citations?

Yes

Are the conclusions drawn appropriate in the context of the current research literature?

Yes

Is the topic of the review discussed comprehensively in the context of the current literature?

Yes

Reviewer Expertise:

Social Determinants of Health; Social Epidemiology; Intimate Partner Violence; Global Health; Mental Health.

I confirm that I have read this submission and believe that I have an appropriate level of expertise to confirm that it is of an acceptable scientific standard.

# Peer review: Reviewer response for version 1

**DOI:** 10.5256/f1000research.23184.r61928

**Published:** 2020-04-14

**Authors:** Teresa Evans

**Affiliations:** 1 Graduate School of Biomedical Sciences and Department of Pharmacology, UT Health Science Center San Antonio, San Antonio, TX, USA

Each topic is discussed comprehensively with well-rounded information provided on the impacts of mental health on undergraduate, graduate, and postdoctoral fellows. Clarification is provided to ensure confusion is not made between discussion of undergraduate data and graduate level data, particularly via tables. Further, the authors provide clear definitions of Depression, Anxiety, and Wellness along with a discussion of the scales used to assess symptomatology. Discussion of challenges faced by graduate students and postdoctoral fellows is comprehensive and well referenced. It is also important to note that the authors provide a strong overview of the data present for medical and health students as well. 

 All statements provided are factual and supported by citations. The review is well written and accessible. Also, all conclusions drawn are appropriate for the literature cited and the state of the field.

Is the review written in accessible language?

Yes

Are all factual statements correct and adequately supported by citations?

Yes

Are the conclusions drawn appropriate in the context of the current research literature?

Yes

Is the topic of the review discussed comprehensively in the context of the current literature?

Yes

Reviewer Expertise:

Graduate trainee mental health, K-12 STEM education, neuroscience.

I confirm that I have read this submission and believe that I have an appropriate level of expertise to confirm that it is of an acceptable scientific standard.
